# Hospital and Institutional News

**Published:** 1919-08-02

**Authors:** 


### HOSPITAL AND INSTITUTIONAL NEWS.

### MINISTRY OF HEALTH.

The Minister of Health has appointed Sir George Newman, K.C.B., M.D., F.R.C.P., as Chief Medical Officer of the Ministry, with status corresponding to that of a secretary of the Ministry. By arrangements between the President of the Board of Education and the Minister, Sir George Newman retains his position as Chief Medical Officer of the Board of Education. Five new posts of "Senior Medical Officer" have been established, with status corresponding to that of assistant secretary. To these the Minister has appointed the following: G. S. Buchanan, C.B., M.D.; Miss Janet M. Campbell, M.D., M.S. (who will also, by arrangement with the President of the Board of Education, act as Chief Woman Medical Adviser of that Board); F. J. H. Coutts, M.D.; A. W. J. MacFadden, C.B., M.B.; and J. Smith Whitaker, M.R.C.S. (who will also act as Medical Adviser to the National Health Insurance Joint Committee). The whole of the rest of the established medical staff of the Ministry will be in one grade to be known as "Medical Officers." They will comprise the remainder of the existing medical staffs of the Local Government Board and of the Insurance Commission, with the addition of new officers still to be appointed as the additional services may require.

### PENSIONED MEN.

We read that there is reason to believe that the Ministry of Pensions has estimated that two thousand beds will be sufficient accommodation for the sick men under their care in the London district. If this estimate is applied to Greater London, then we fully agree with the correspondent of the *Times*, who points out that there are many more pensioners than this who will be requiring hospital treatment of some sort at any given time, at least during the periods immediately ahead of us. It is a most difficult problem efficiently to deal with these men, particularly orthopædic cases, and the chronic systematic infections which are not only unsuitable for haphazard distribution throughout our smaller hospitals, but require very specialised knowledge in their medical attendants. Well-distributed out-patient clinics, reserved beds in outlying hospitals, military and civil, or concentration upon one or two of the larger London hospitals are all suggested as possible measures, but individually each plan has its obvious shortcomings and is alone open to much criticism. The men themselves are deserving of very special consideration, apart from their relation to the health of the community as a whole, and we are certain that the Ministry will spare no pains to make whatever scheme is finally adopted absolutely thorough and efficient, in this case radically changing, if necessary, many of our formerly accepted methods of both in- and out-patient organisation. The majority of these pensioners represent very specialised types of malady, and, as the *Times* points out, it is a question of specialist service.

### PLAGUE AT AVONMOUTH.

The Ministry of Health make the announcement that on arrival of the s.s. *Framlington Court* at Avonmouth from Montreal on the 22nd instant one of her officers who was ill was removed to the isolation hospital and has been certified to be suffering from bubonic plague. Another officer with suspicious symptoms was taken to hospital the following day, and he is also suffering from the disease. The vessel came from Alexandria to Montreal and called at Sydney, Nova Scotia, on her voyage to Avonmouth. She is being detained by the Bristol Port Sanitary Authority, and all precautions have been taken. The circumstances are being fully-investigated by one of the medical officers of the Ministry.

### THE MESOPOTAMIA MUDDLE EMULATED.

The Secretary of State for India has appointed a Committee to inquire into the administration and organisation of the Army of India. This inquiry will no doubt include investigation into the alleged breakdown of the medical arrangements of the campaign still in progress on the Indian frontier. It is an open secret that there has been, to a limited extent happily, a repetition of the scandals which attended the early months of the Mesopotamian advance; and it seems extraordinary that, after the disclosures of 1917, the medical part of the organisation of the Army on active service should, even partially, have broken down. In June there was a serious cholera epidemic at the front, apparently due to defective arrangements for water supply. A leading article in the *Times* states that the news of this outbreak was suppressed at the time, in deference to those "military reasons" which are used to cover up so many shortcomings. The epidemic is now over, and in the middle of July it was stated that there had been 1,663 cases and 566 deaths, chiefly among the Indian "followers." The British deaths numbered six officers and fifteen other ranks. The article quoted calls attention to the probability that the Indian military authorities issued no statement on the subject until the leading English newspapers in India began to publish disturbing articles.

### INDIAN HOSPITAL INSUFFICIENCIES.

It is alleged that in one hospital, serving the frontier campaign, there were no sheets, towels, or blankets, no soda water or ice, no chairs and no provision for washing; at one time, even bandages gave out. The condition of the rooms is stated to have been indescribable, they were never cleaned, the beds touched each other, sick and convalescent cases were intermingled, and the food was poor and insufficient. The excuses advanced in defence of the Government in India are that "transport difficulties hampered medical supply in May and early June," and that the campaign "was not expected." As the *Times* says, there was a time when the advanced divisions in North-West India were always practically on a war footing and it would not have been possible for it to have been pleaded that any frontier campaign was "unexpected." The inference is that the defects revealed by the Mesopotamian Commission have never been properly rectified, and their lessons are unheeded; no doubt the new Committee will in course of time be able to bring home responsibility for the shortcomings and those persons chiefly involved will receive promotion, if not in their present service, in some other where their inefficiency will have free play.

### LOCAL ANÆSTHESIA.

An account reaches us of a so-called new method of local anæsthesia which is described as follows. Before making incision, a dry sterile scalpel is dipped in pure phenol and the point of the back of the scalpel is passed over the intended line, marking it out with the liquid. A little later the scalpel is again dipped and the skin incised very slowly and gently. As depth is gained so more phenol is applied to each successive layer as a film on the knife edge, thus producing local anæsthesia. Professor Bruschi, of the chief military hospital, Milan, reports three thousand completely successful cases, the operations being described as absolutely painless. The extreme simplicity of the method makes a strong appeal, but it must be remembered that such incisions will not close so rapidly as those made with an ordinary sterile instrument, and the method appears to be absolutely contraindicated where primary union and suture are desired. Certainly in local septic foci, where drainage by gauze or tubes is needed and closure is advantageously delayed, there appears to be a possible and most useful application. Absorption and toxic effects must be considered, but these, so the authors tell us, have given rise to no serious complication.

### CANCER RESEARCH.

At the recent meeting of the General Committee of the Imperial Cancer Research Fund, the annual report made mention of the fact that the effect of war conditions on the cancer mortality figures of the whole country was first apparent in the national statistics for the year 1915. At that time there appeared a great and sudden increase in the cancer death-rate in males. The rate for females was, however, unaltered, and when the necessary correction was made by allowing for the departure of large numbers of young males upon military service, the apparent rise in the first-mentioned figure also disappeared. Further, it was explained that the financial position of the Committee, owing possibly to the diminished expenditure due to decreased opportunity for work during the past years, was such that under the circumstances there was no need to make an appeal for funds at the present time.

### A CIRCULATING LIBRARY FOR HOSPITALS.

We have recorded in the past the few attempts that have been made to affiliate hospitals with the public libraries of their nearest towns, and some of these schemes were for a time at least successful. Now that the War Library, which from its central depôt, Surrey House, Marble Arch, has mainly served its purpose, Mrs. H. M. Gaskell, C.B.E., has sketched its history in a pamphlet which has a practical interest for hospital managers. It is issued by the Red Cross Society. With the help of Dr. C. Hagberg Wright, of the London Library, it is hoped to continue the War Library permanently, so as to provide a depôt in London which may circulate books to all civil as well as naval and military hospitals. The present large stock of books will be something more than a nucleus, and the public will be invited periodically to contribute books and magazines, preferably *not* parish magazines, which are too local in scope and interest. A board of five trustees, one of whom is to be a professional librarian, is to meet not less than ten times a year, and quarters have yet to be found in an accessible and inexpensive part of London. The staff proposed is to consist of a librarian, his assistant, a boy, a packer, and a maimed man, whose wife would do the cleaning and live on the premises. If the Post Office can be persuaded to extend even a small share of its war-time assistance, the Red Cross Library should be a welcome institution.

### A POOR LAW EXPERIMENT.

The more humane treatment of the poor in institutions is shown by an experiment Southwark Board of Guardians are trying for a month. They have decided to allow inmates in their institutions over 60 years of age leave one day during the week in addition to their Sunday leave, whilst those under 60 are to be allowed leave once a fortnight instead of once a month, as at present. In a large institution with close on 600 inmates there may be some difficulty in carrying out this new arrangement, but the success depends entirely on the inmates themselves. Often when allowed out they are treated to intoxicants by their friends, with the result that they return in a condition that is not conducive to good government and order. It is this danger that may threaten the success of the scheme for the benefit of the inmates.

### SHOULD CHILDREN BE PUNISHED FOR SMOKING?

In his report on the medical inspection of Liverpool school children, Dr. Hope calls attention to the increase in the number of cases of scabies which are twice as numerous as those recorded in 1916. This may be merely a tribute to the attention now paid to medical examination, but if the extent of the disease is now known, it is desirable that the Army methods of baths, disinfectants, and trained attendants should be followed. Without them cases which should be treated successfully in ten days linger for several months and no doubt spread the infection. The cost, Dr. Hope thinks, would be met out of the increased Government grant for which the centres would then be qualified. Juvenile smoking is still reported to be prevalent, and Dr. Hope wishes that a breach of Section 40 of the Children Act should have the sanction of a penalty. We are against the multiplication of regulations and penalties affecting the daily life of the people.

Smoking is bad for children, but to punish them or their parents for an error which is not a crime would be a form of regimentation more congenial to Prussia than to ourselves.

### AN IMPORTANT DEVELOPMENT AT WINCHESTER.

At the instance of the Hampshire County Council, a maternity department has been added to the Royal County Hospital. When the Notification of Births Act of 1907 became compulsory in 1915, the County Council became responsible for maternity work and child welfare. The Public Health Committee, whose request to the voluntary hospitals for assistance was generously declined on the ground of soldiers' claims, found a supporter in Mr. Henry Nicholl, an old friend of the Royal County Hospital. Thanks to his aid, its old rules, which coupled cases of maternity with those of "disordered intellect," and where the County Council agreed to pay for necessary alterations, Mr. Nicholl supplied the bulk of the equipment. The County Council will maintain the new department, and Dr. Lyster, Medical Officer of Health, hopes that it will treat three hundred cases a year. Payment will be proportional to the means of the patients. By the beds are cots—the customary cradle at the foot is thus improved upon. The staff consists of Dr. James Smythe, the obstetric medical officer, Sister Ball, who qualified at the Rotunda Hospital, Dublin, so that nurses can be trained for the C.M.B. certificate. At the opening Mr. Nicholl said that he had contrasted the State hospitals of the continent with the voluntary hospitals of England, and had become convinced of the superiority of the latter. Dr. Fuller England and Dr. Lyster also spoke, and both believe that the new department will be the centre of a growing work, until it will become as normal for women to enter hospitals for their confinements as it is now for them when an operation is in view.

### A MIDWIVES ACT EXPERIENCE.

The Midwives Act, 1918, imposes on local supervising authorities the payment under conditions of the fees of medical men called in by midwives. The Act also empowers County Councils to recover such fees from the patient, unless the patient is too poor to pay the fee. The Middlesex County Council recently declined to make claims until inquiry was made into the financial circumstances of all the cases. Now the Council reports that the Act came in force on January 1, and for the six months ended June 30 ninety-five applications for payment had been received from doctors. During the same period 529 notifications of sending for medical aid were received from midwives. From this it would appear that in 82 per cent. of the cases in which a doctor had been summoned, payment had been made to the doctor direct by the family, or else that he did not claim a fee from the County Council. It follows that the majority of cases in which application is made are cases of poverty, as the amount of fees so far received from patients by the County Council shows. Out of ninety-five cases the Council decided in sixty cases not to attempt to obtain repayment. In the six months the total fees paid to medical practitioners amounted to £69 12s. The amount received was £9 10s.

### THE LAST "GAZETTE" OF 3rd LONDON.

The forty-sixth and final number of the *Gazette* of the 3rd London General Hospital is now issued, price 1s. It is what is known in the trade as a double Peace number, even more generously illustrated than usual, though necessarily only a few of the original contributors and early draughtsmen are represented. We say goodbye in it to such familiar names as Ward Muir, Noel Irving, Helen Nightingale, S. B. de la Bire, Derwent Wood, Vernon Lorrimer, C. R. W. Nevinson, H. Hemsley, and the rest of these humorous or sentimental chroniclers of the War. To remind us of their collective achievement, no illustrations are more interesting than those of the old company clothing store, which the brush of S. G. J. Coates converted into a Roman Catholic chapel garnished with wall-paintings in panels that, so far as they can be seen in reproduction, have distinct artistic value. Any criticism of ours, whether of praise or not, has been forestalled, however, as readers will see who turn to Cpl. J. H. Dowd's illustration on p. 325 of the *Gazette*, which a previous number of The Hospital inspired. We once begged the contributors to this the last number to "let themselves go." They are depicted carrying out The Hospital's advice, with the inscription "and they did," a fitting epitaph! As the former C.O. remarks in valediction: "if Ward Muir and Noel Irving, the past and present editors . . . could be persuaded to start a paper of this character for a wider circulation, and could secure the help of their war-time contributors, its success would be assured." This is true; but until they do so, The Hospital will continue to welcome, as it has done in the past, contributions from them wherever they may be scattered.

### AT LEEDS INFIRMARY.

At a meeting of the Special Election Committee for the appointment of honorary officers, held on Friday, July 25, 1919, the following appointments were made:—Dr. George W. Watson, M.D., F.R.C.P., was elected honorary physician to fill the vacancy caused by the retirement of Dr. Churton, after forty years' service. Dr. Watson for eight years has occupied the position of honorary assistant physician. Mr. L. R. Braithwaite, M.B., Ch.B., F.R.C.S., was elected surgeon in charge of out-patients, having held the position of assistant honorary surgeon for ten years. Mr. Harry Lee, B.A., M.B., B.C., F.R.C.S. (Eng.), was elected honorary ophthalmic surgeon. Dr. J. Le Fleming Burrow, M.B., Ch.B., M.R.C.P., was elected honorary assistant physician to fill the vacancy caused by the election of Dr. Watson to the full staff.

### THE OPEN GRATE.

Timely reference to the fact that "the price of coal will have a direct influence on the health of the community" is made in the annual report of

Dr. W. J. Lewis, M.O.H. for the Pontardawe rural district, which embraces within its area a little nest of typical South Wales mining villages. "Much has been said against the wastefulness of open grates," comments Dr. Lewis, "but I am not sure that the extravagance is not worth it in comfort and health. . . . . The attraction of public houses is to some extent due to their blazing fires. Nothing is so comforting and restorative to a tired man as radiant heat from a red fire in a well-ventilated room." Dr. Lewis adds that radiant heat has not only restorative but heating powers. He is not the only M.O.H. who looks forward with apprehension to a winter in which the high price of coal may make it practically prohibitive to the poorer classes and to many aged and sick people.

### AN EXTRAORDINARY FINANCIAL HISTORY.

For the third, and only for the third, time in nearly two hundred years, Worcester Infirmary has been notified of a coming legacy of five figures. This is therefore a rare and welcome prospect, which will be accomplished under the will of Mr. James Hugh Allan. The hospital has had an extraordinary financial history. The number of its beds has fluctuated from the first. When extensions were not being made, wards were being closed. Needless to say, the latter policy accomplished nothing except in the withdrawal of public confidence which always follows such a disastrous step. In 1900, for example, the annual subscription list was lower than it had been since 1860. In 1913, before the war, the beds were reduced to seventy, and such of the staff as could be spared were dismissed. Let us hope that this big legacy will encourage a forward policy. It is the work which brings the support in the long run.

### A BANKRUPT IRISH HOSPITAL.

Hospitals occasionally do close their doors, notwithstanding the false alarms by which some of them seek to flourish. It has been stated that the Hardwicke, the Richmond, and the Whitworth hospitals Dublin, which are managed by a common board, are reduced to such straits that they cannot pay their tradesmen's bills. The Dublin Corporation is making an appeal to the Irish Government, which has subsidised the joint board since 1856 with an annual grant of £7,600. The cost per bed has risen from £64 to £119 since the war, and the hospitals are said to have no other funds than the grant referred to which has passed into the possession of the bank to reduce the overdraft. The board seems to regard the case as hopeless, and all its members have resigned. Since they are appointed by the Lord Lieutenant under the terms which condition the Government grant to the three Houses of Industry hospitals, the Government cannot stand aside, even if it contents itself with the appointment of a new board, supposing that new men will come forward to accept a trust which has fallen into such a bad state. The problem affects Dublin Corporation because the hospitals of the city are flooded with patients from the country, which is badly off for local hospitals. At the same time, the Irish Government, in making the old grant, accepted responsibility, and the value of money has declined.

### VOLUNTARYISM IN BIRMINGHAM.

We are sorry to learn that the circular issued by the Lord Mayor of Birmingham for new subscriptions to the hospitals of the city has met with a response from a relatively small circle of supporters only. It is to be followed by a personal canvass, which will no doubt be more productive. The sum raised, however, is far from the end of the matter. To badger people into giving is hardly to rely on voluntary support; and though it is better than to tax them, because the evil effects of bureaucratic control are avoided, it means that the voluntary principle is not the success which it ought to be. Progressive Birmingham is badly behind other cities in the number of hospital beds, and even these cannot be maintained properly. The problem, therefore, is not only to raise sufficient funds to provide the new beds and to maintain them afterwards, but to save the population in spite of itself from the conditions which the Ministry of Health will necessarily impose in return for its assistance. The problem of ways and means oppresses voluntary hospitals so heavily that their managers need to be men of exceptional intelligence to see that something more than ways and means is expected from them. Charity, the voluntary principle, will become something less than commercial money-getting, if it is not something more. Hospital men must be apostles as well as managers, and believe in the principle if they are to be eloquent and even convincing on its behalf.

### THIS WEEK'S DRUG MARKET.

The market is being watched with close attention, so that, although the volume of business is not large, the market is far from being uninteresting. In the majority of cases it is really difficult to know what to do—whether first to buy from hand to mouth or to take in stocks to cover any lengthy period. Some hold one view, others another, and the outlook is very uncertain. The tendency generally is towards higher prices, but the factors on which its upward movement is based are by no means settled. So long, however, as the restrictions remain on imports it is almost certain that the drugs affected by these restrictions will not move downwards in price, unless it is in exceptional cases. The higher value of carbolic acid affects some other products, as, for instance, salicylic acid, which has advanced in price. Values of aspirin, phenacetin, phenazone, antipyrin, and hexamine are firmly maintained. There is a lull in the upward movement of menthol, but the price is very high. Japanese refined camphor is a quiet market, and there is a slight falling off in value. Star aniseed oil is moving upwards. Cascara sagrada is dearer. Clove oil has advanced in sympathy with cloves. Senega is dearer. Bromides have taken a turn for the better, and we may see higher prices.

